# Residents’ and preceptors’ perceptions of the use of the iPad for clinical teaching in a family medicine residency program

**DOI:** 10.1186/1472-6920-14-174

**Published:** 2014-08-20

**Authors:** Douglas Archibald, Colla J Macdonald, Judith Plante, Rebecca J Hogue, Javier Fiallos

**Affiliations:** 1 Department of Family Medicine, University of Ottawa, 43 Bruyère St., Ottawa, ON K1N 5C8, Canada; 2 C.T. Lamont Primary Health Care Research Centre, Bruyère Research Institute, 43 Bruyère St., Annex E, Ottawa, ON K1N 5C8, Canada; 3 Faculty of Education, University of Ottawa, 145 Jean-Jacques-Lussier Private, Ottawa, Ontario K1N 6N5, Canada; 4 Pembroke Regional Hospital, 705 Mackay St., Pembroke, Ontario K8A 1G8, Canada

**Keywords:** Tablet, Handheld computers, Preceptor, Medical applications, ELearning

## Abstract

**Background:**

As Family Medicine programs across Canada are transitioning into a competency-based curriculum, medical students and clinical teachers are increasingly incorporating tablet computers in their work and educational activities. The purpose of this pilot study was to identify how preceptors and residents use tablet computers to implement and adopt a new family medicine curriculum and to evaluate how they access applications (apps) through their tablet in an effort to support and enhance effective teaching and learning.

**Methods:**

Residents and preceptors (n = 25) from the Family Medicine program working at the Pembroke Regional Hospital in Ontario, Canada, were given iPads and training on how to use the device in clinical teaching and learning activities and how to access the online curriculum. Data regarding the use and perceived contribution of the iPads were collected through surveys and focus groups. This mixed methods research used analysis of survey responses to support the selection of questions for focus groups.

**Results:**

Reported results were categorized into: curriculum and assessment; ease of use; portability; apps and resources; and perceptions about the use of the iPad in teaching/learning setting. Most participants agreed on the importance of accessing curriculum resources through the iPad but recognized that these required enhancements to facilitate use. The iPad was considered to be more useful for activities involving output of information than for input. Participants’ responses regarding the ease of use of mobile technology were heterogeneous due to the diversity of computer proficiency across users. Residents had a slightly more favorable opinion regarding the iPad’s contribution to teaching/learning compared to preceptors.

**Conclusions:**

iPad’s interface should be fully enhanced to allow easy access to online curriculum and its built-in resources. The differences in computer proficiency level among users should be reduced by sharing knowledge through workshops led by more skillful iPad users. To facilitate collection of information through the iPad, the design of electronic data-input forms should consider the participants’ reported negative perceptions towards typing data through mobile devices. Technology deployment projects should gather sufficient evidence from pilot studies in order to guide efforts to adapt resources and infrastructure to relevant needs of Family Medicine teachers and learners.

## Background

A wide variety of applications for tablet computers are available for needs ranging from the most basic level of medical undergraduate education to specialist care delivery. Undergraduate medical students have used handheld computers to access drug references and clinical medicine handbooks [1,2]. Internal medicine residents have use Apple iPads (Cupertino, California) to access medical records, online publications and paging resources [3]. Residents from orthopedic surgery programs access Electronic Medical Records (EMR), orthopedic literature, prepare for Orthopedic In-Training Exam (OITE) and use apps to keep notes [4]. Radiology residents use apps to review anatomy atlases, case discussions, online articles and books [5].

Successful implementations of tablet computers in medical education programs have shown to improve clinical decision making in undergraduate medical students [6], increase efficiency of internal medicine residents’ rounds [3], contributed to “overall teaching quality” of a rotation of anesthesia for orthopedics [7] and improved performance of medical students on national exams [8]. However, the introduction of technology in education deserves careful consideration as the students might have different preferences and levels of experience with each type of technology and institutions might not always provide appropriate supporting infrastructure [9]. A recent study shows that there is a high frequency of tablet computer use among medical students [10], which suggests that the number of educational programs attempting to adapt to these modern students’ behaviors, will increase through time. Several medical schools have already incorporated the use of tablet computers in their curriculum [11] and the trend is on the rise as manufacturers increase usability and capability of devices.

As technology changes with time, so are the educational needs of medical students which have motivated a shift in focus from rotation-centric to competency-based education. A clear example of this evolution is the initiative of the College of Family Physicians of Canada (CFPC) that promotes the Triple C Competency-based Curriculum (Triple C). This curriculum is described as “comprehensive; focused on continuity of education and patient care; centered in Family Medicine” [12] and is intended to ensure that trainees graduate as skillful and knowledgeable competent physicians. All Canadian medical schools are in the process of adopting the new competency-based family medicine Triple C curriculum. This model has already started to affect the design, implementation and accreditation of all residency programs in Canada including the assessment of residents. Mobile devices such as tablets could be of a great value in facilitation of the teaching and learning process in competency-based curricula [13].

The Department of Family Medicine at the University of Ottawa has developed a framework (http://familymedicine.uottawa.ca/curriculum-framework/) to guide the adoption of a Triple C competency based curriculum, that incorporates the curricular elements of family medicine. These elements include nine domains of clinical care, the four pillars of family medicine [14], and the CanMEDS –FM [15]. All elements of the curriculum framework, including detailed competencies for each clinical domain were available to all participants in this study.

Our research focuses on identifying how tablet computers are used in the Family Medicine residency and how they contribute to teaching and learning under the Triple C Competency-based Curriculum. More specifically, the objectives were twofold: (1) evaluating the experience of specialist and family medicine preceptors, and residents using tablet computers to implement and adopt the new family medicine curriculum; (2) evaluating the extent to which family physicians and residents access tablet applications (apps) through their tablet in an effort to support and enhance effective teaching and learning.

To the best of our knowledge this study is the first attempt to describe attitudes and perceptions of Family Medicine residents and preceptors regarding the use of iPads in their clinical teaching/learning work. This article presents a mixed methods approach where participants report how they perceive: the experience of using the iPad to access the Triple C Competency-based curriculum and its assessment resources; the ease of iPad use for multiple activities; its portability; usefulness of apps and resources; and its overall contribution in the teaching/learning process. Some conclusions that inform planning of technology deployment for teaching/learning enhancement are drawn from the results.

## Methods

Participants in this study were preceptors and residents from the Family Medicine program at the Pembroke General Hospital located in Pembroke, Ontario, Canada. Prior to the commencement of the pilot study the preceptors and residents completed a questionnaire designed to capture information about their computer skills, experiences, attitudes towards computers and preferences for medical apps. The collected data were used to aid the design of training modules within the pilot study and to guide the selection of medical apps to be installed. This study was reviewed and deemed exempt by the Ottawa Health Sciences Research Ethics Board and the Pembroke Regional Hospital Research Ethics Board.

In February 2012, the eight-month pilot study was started with a group of 25 participants including 20 preceptors and 5 residents. 11 preceptors were family physicians and 9 were from other specialties. All participants were given 3G enabled iPads and received data plans to avoid limitations to access to online resources. They were also loaded with medical and general apps such as drug references, and calculators. See Table 1 for a list of medical and general apps that were loaded onto participant’s iPads. Participants were encouraged to find additional apps and resources by themselves and use them for their clinical teaching and learning activities.

**Table 1:** Medical and general apps loaded onto iPads

**Pre-Loaded Apps**	**Resident**	**Preceptor**
RxFiles®	x	
iDoctor®	x	
LexiComp® databases	x	x
Lab Results®	x	x
Scat2®	x	x
My Healthy Waist®	x	x
Calculate®	x	x
Evernote®	x	x
DropBox®	x	x
iBooks®	x	x

A half-day educational workshop was provided to support participants in becoming more confident with using the tablet, its installed apps and accessing the Triple C curricular framework [12], objectives and evaluations. Learners were oriented the basic functions of the iPad such as downloading apps from the Mac App Store, using email and other common communications functions such as Dropbox. After the orientation learners were given the opportunity to explore their iPad as four workshop facilitators with extensive experience using iPads answered questions participants had about their new devices.

In addition to the introductory workshop, there were two more workshops provided by one of the participants who was identified as an early adopter of the iPad. Furthermore, the lead evaluator of the pilot study periodically met with the participants over a six month period. These personal meetings gave the participants an opportunity to ask questions, receive additional coaching and feedback and voice any concerns about the study. Participants’ experience was evaluated at the beginning, at four months and at the end of the study.

The study used a sequential explanatory mixed methods research design [16]. This allowed results from a quantitative data collection first stage to guide the design of questions to collect qualitative data in a second stage. The qualitative component adhered to the RATS guidelines [17] which states that study questions be relevant; the qualitative method be appropriate; procedures be transparent, and; the interpretive approach be sound. In the first stage, quantitative data were collected through a mid-point validated online survey. This survey was a modified version of the CE + HD iPad Initiative Student Survey [18]. It contained closed-answer items intended to capture usability patterns of participants, degree of collaboration enabled by the device, problems encountered when using it in the study setting and the perceived impact of the iPad in their teaching/learning. These data were analyzed by calculating descriptive statistics for the learners’ responses on the closed-answer survey items. Differences in perceptions between residents and preceptors were assessed through non-parametric hypothesis tests due to the small size of the sample which precluded assuming data were normally distributed. Qualitative data were collected through audio-recorded focus groups interviews with all participants. Interviews were transcribed verbatim. The purpose of the focus groups was to (a) provide participants with the opportunity to elaborate on and increase our understanding of issues and concerns identified in the survey data; and (b) identify additional themes not captured by the survey. The questions for the focus group were framed by the constructs of the CE + HD iPad Initiative Study Survey: including effectiveness and effectiveness of using the iPad for clinical teaching (see Additional files 1 and 2).

Qualitative data were analyzed using a content analysis approach. The research questions arising from the quantitative analysis were used as a framework from which to search for themes and meanings emerging from the data. The categories, themes and patterns that emerged in this process were evaluated for their credibility. Agreement on the categories was reached by one the investigators and his research assistant. At the same time, the evaluators searched through the data for disconfirming instances and alternative explanations. Direct quotations were used throughout the reports in order to preserve the voice of the participants.

## Results

Four residents and 13 preceptors completed the mid-evaluation survey (68% response rate) while 9 preceptors and 4 residents participated in the focus groups. One of the residents completed his training shortly after the study began. The 4 remaining residents all completed the survey and participated in the focus groups. Of the 17 participants, ten previously owned an iPad before participating in this study, and five had previously owned or currently own a smart phone (iPhone or Blackberry). The following section summarizes main results which have been organized into five categories related to curriculum and assessment; ease of use; portability; apps and resources; and perceptions about the use of the iPad in teaching/learning setting.

### Curriculum and assessment

The Family Medicine Curriculum Framework was included as pre-loaded content of the iPad tablets. This electronic version of the framework provided links to the Family Medicine Learning Objectives and to the daily resident feedback form (field note).

About 41% of participants reported to use the iPad to access the curriculum. This was a lower percentage compared to the proportion of participants using the iPad for activities such as email (88%), medical apps (88%) and access to medical databases (88%).

Most of the comments about using the iPad to access the curriculum focused around assessment, specifically on the field note that preceptors should be using to formatively assess their residents. Only two out of 17 participants reported using the online field note through their iPad and 67% of respondents stated that access to field notes was problematic. Additionally they remarked that is was too detailed and its completion required an overwhelming effort. Part of the reasons behind this low utilization of resources was the fact that the online version of the Family Medicine Curriculum for tablet computers was at a developing stage.

Although all participants were positive regarding the adoption of the field note and remarked that an electronic field note was imperative in order to collect feedback through a central database, they felt that in order to increase the use of electronic versions of the curriculum framework and field notes they both needed to be more user-friendly.

### Ease of use

**Figure 1:**
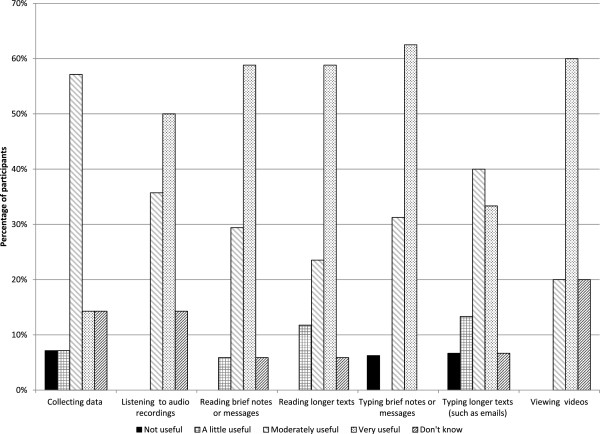
Usefulness of iPad for teaching/learning activities.

About 91% of participants agreed that the iPad was easy to use, however, users reported to have different preferences in terms of what they accessed and found helpful. Confident iPads users expressed they used the device to read a medical history, access multimedia resources and take notes at meetings. However, most preceptors stated only used the iPad when there was no desktop computer available.Figure 1 shows the distribution of participants’ opinions regarding the usefulness of the iPad for specific activities. Activities where the iPad generated an output of information (i.e. listening to audio, reading texts and viewing videos) were judged slightly more useful compared to input of information activities (i.e. collecting data, typing text). One preceptor commented: “It’s big enough to see well, but the keyboard is too small to easily type on. It’s okay for a little message but if I am seriously going to write something I don’t like this, It’s too small….” (Preceptor 3).

### Portability

The majority of participants agreed that the iPad was a portable device. However, not all physicians found they were using the iPad in their clinical work or teaching. The following specialist preceptor indicated in an email that using the iPad in the Emergency Room did not work: “As a doctor in the emergency department, I am always on the go, doing different things and multitasking. I am zig-zaging from patient to patient. It is not practical to carry a computer with me in the rooms to see patients, especially when the patients are more critical, I need both my hands” (Preceptor 4).

Participant’s opinions about the portability of the iPad varied. Some participants stated the iPad was not portable enough since it does not fit in their pocket. However participants expressed the size of the iPad is convenient to carry around to show videos and images to patients and residents. It was also expressed that the iPad eradicated the need to carry heavy books since it provided access to large amounts of information (e.g. RxFiles) in a light weight device. Others participants mentioned that the portability of the iPad provided an option to access information systems when desktop computers at nursing stations were not available. However preceptors pointed out the likeliness of theft and loss as down side of the iPad’s portability. In addition, participants noted it is inconvenient to carry the iPad into isolation rooms due to contamination issues.

### Apps and resources

Residents reported the most beneficial app was the *RxFiles*, which is the Canadian drug data base used by residents at the University of Ottawa. However, one of the preceptors noted that *RxFiles* is moderately searchable since it is more like a “digital paper copy” and proposed alternative resources such as the *Sanford Guides*©, which provide enhancements to improve search ability.

By far the most popular app for preceptors was the LexiComp app. A preceptor felt that drug interaction information was more reliable on Lexicomp compared to the EMR’s capability. From the apps found by participants, residents considered the Show Me app to be very popular. It can be used as an audio recording and also a whiteboard. One resident described how she used it. *“*It takes an audio recording and I can free hand, so I have found that useful because with an hour lecture I can audio tape. I am not very organized with papers, so this has been very helpful” (Resident 2).

The availability of numerous apps for each type of need made it difficult for users to determine the best options to install in their device. Less experienced iPad users looked for advice from experienced peers to aid in their selection. More confident iPad users installed additional apps to enhance their clinical and teaching/learning performance. In most cases, preceptors found the self-installed apps more useful than pre-loaded apps. These included for example “Show Me” an app that displays a white board, audio and video, and “iAnnotate” which is an app that can edit PDFs.

### Perceptions about the usefulness of the iPad in teaching/learning setting

**Figure 2:**
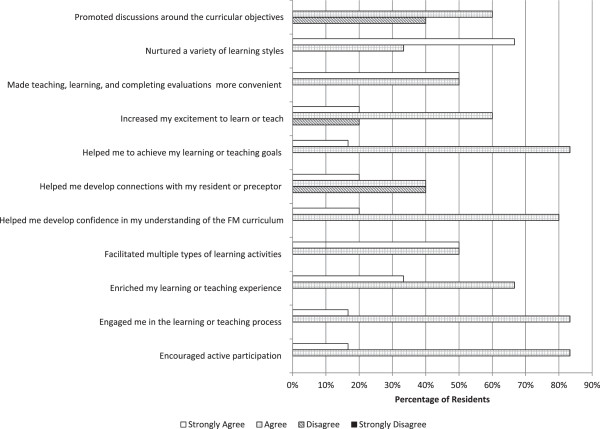
Perceptions of residents regarding the use of iPad in Family Medicine training.

**Figure 3:**
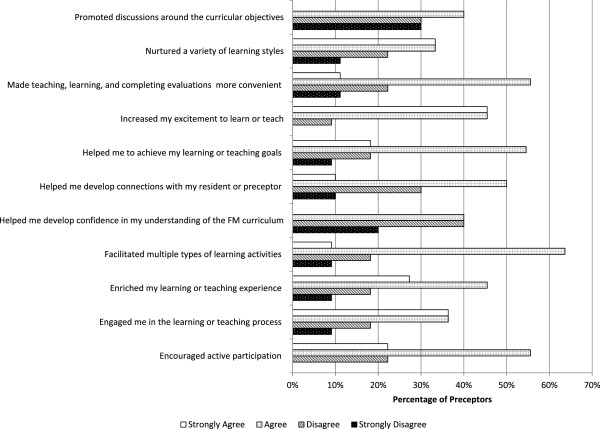
Perceptions of preceptors regarding the use of iPad in Family Medicine training.

Results from residents and preceptors were analyzed separately in order to identify possible differences between groups. Non-parametric Mann–Whitney tests showed that perceptions between residents and preceptors were not significantly different, except for the item labeled “Helped me develop confidence in my understanding of the FM curriculum” (residents’s mean (SD) = 3.2 (0.58), preceptors’ mean (SD) = 2.76 (0.89), p = 0.04) indicating that perceptions of residents in this item were more positive than for preceptors.Figures 2 and 3 show slight evidence of less favorable perceptions from preceptors regarding the use of the iPad in educational activities in most survey items. Results from preceptors and residents as a whole indicate 76% of the ratings for these 11 items fell under the “agree” or “strongly agree” options, meaning the majority were positive about the contribution of the iPad to effective teaching and learning.

## Discussion

The lack of a friendly interface to search the curriculum and the inability of typing on the field note were cited as discouraging difficulties. This highlights the need to ensure not only resources are available but they must be at a mature operational stage that facilitates navigating through contents regardless of the type of device.

In terms of the ease of use, respondents considered the iPad was more useful for activities involving output of information than for input of data. There was a clear, although not unanimous, preference for desktops and laptop computers when typing a long text was required. Overall, it was evident that participants’ responses regarding the ease of use of mobile technology were heterogeneous, which motivates the need for an increased awareness of the diversity of baseline needs, capabilities and interests of users when initiating an educational program of this kind. This is consistent with findings reported in other studies [9,19] emphasizing the importance of acknowledging the diversity of user characteristics.

The portability of the iPad was considered an important attribute that contributed to quick access to learning resources making it easier to provide a classroom setting in almost any location. The iPads size, weight and capacity to store large amounts of data and connect to online information sources and apps makes it a great tool for enhancing collaboration between preceptors and residents, aiding medical decision making at the point of care and facilitating interactions with patients. One preceptor in particular introduced the “Show Me” app to residents and other faculty. This app was quickly adopted by participants to show quick diagrams with accompanying audio for in the moment teaching. However, not all situations provided an advantage for the iPad’s portability due to infection prevention measures, risk of theft and loss or simply because some participants considered the size not small enough to carry around.

Apps were considered one of the most important resources that helped residents and preceptors in clinical work; however, selecting the best set of apps was not straightforward. In the search for a better pool of resources, more enthusiastic iPad users led continuing education workshops for peers to provide reviews of apps and share recommendations on how they could enhance teaching and learning.

Perceptions of participants were generally favorable regarding the contribution of the iPad to teaching/learning goals (See Figure 3). These results should be interpreted cautiously since there is the possibility of an overestimated enthusiasm “hype” with iPad use, typical of initial stages of technology deployment [20]. Differences in opinion about the iPad’s contribution to teaching and learning might also be a product of the variability in computer proficiency level, usefulness of self-selected apps, or a combination of both. This difference might also be explained by the residents generally being more open to using mobile technology for learning and their willingness to make time to learn how to use it. Yet another explanation may be that preceptors found the iPad challenging for teaching. As there is some evidence from the surveys that preceptors’ perceptions are less positive compared to residents’ which motivates further research to unveil the factors explaining this difference.

The limitations of this study were the small sample size and the recruitment of participants from a single institution in a rural community setting. Although these limitations precluded stronger generalizations and a richer set of participants’ opinions, most observations derived from the quantitative analysis were consistent with qualitative findings that confirmed them and provided explanatory insights.

An additional limitation was the inability to access EMRs using the iPad which would have provided an additional source of information regarding the usefulness of iPad in clinical work.

## Conclusions

The results of our findings have provided an insight on the experience of specialists and family medicine preceptors as well as family medicine residents using a tablet to implement and adopt a new family medicine curriculum. In addition, we also gained a better understanding of how iPad resources are used by physicians in the clinical setting.

In addition to the availability of an interface that allows effective navigation through electronic resources of the curriculum and an infrastructure that supports reliable connectivity, efforts should be made to reduce variability of computer proficiency level of users in order to maximize the performance enhancement opportunities provided by mobile devices. The effects of this variability might be mitigated by promoting continuing education workshops led by residents and preceptors with more advanced skills in using the iPad. These workshops would facilitate sharing their knowledge on how to select apps and resources and how to use them in clinical teaching/learning, thus reducing the gap between expert and novice iPad users. Future research should establish a minimum competency standard for both residents and preceptors. This is important to foster better adoption of any technology.

It can be inferred from the study results that the small size of the mobile devices discourages the typing of data, therefore, the design of any data-input electronic form should be efficient enough to collect most valuable information with minimal effort.

This study provides direction for future research for exploring the effectiveness and impact of tablets in clinical teaching and learning. This study suggests an area of future research may be to explore the use of tablet computers for improving patient education and inputting electronic orders and prescriptions in a larger sample of users.

We believe it is worthwhile pursuing the tablet as a production, teaching and learning tool if the Family Medicine Department supports the deployment of technology adapted to residents and Faculty staff needs. The identification of these needs is not a straightforward task and demands obtaining reliable information from pilot studies findings to better understand the potential effects of introducing new technologies.

## Competing interests

The authors declare that they have no competing interests.

## Authors’ contributions

DA conceived and designed the study, collected data, analyzed data. CJM, JP and RH contributed to the conception and design of the study. JF contributed to data analysis. All authors contributed to the drafting, and revisions of the manuscript. All authors read and approved the final manuscript.

## Pre-publication history

The pre-publication history for this paper can be accessed here:

http://www.biomedcentral.com/1472-6920/14/174/prepub

## Supplementary Material

Additional file 1 **Focus Group Questions for Preceptors.** (Modified questions from the Research & Evaluation Team, OIT, University of Minnesota). Click here for file

Additional file 2 **Focus Group Questions for Residents.** (Modified questions from the Research & Evaluation Team, OIT, University of Minnesota). Click here for file
