# Supplementary material for: Residents’ and preceptors’ perceptions of the use of the iPad for clinical teaching in a family medicine residency program
Source: BMC Med Educ. 2014 Aug 20;14:174. doi: 10.1186/1472-6920-14-174 (PMC4236569; doi:10.1186/1472-6920-14-174)
Supplement: Additional file 1 — Focus Group Questions for Preceptors. (Modified questions from the Research & Evaluation Team, OIT, University of Minnesota). [file 1472-6920-14-174-S1.docx]

# Additional files

### Additional file 1 – Focus Group Questions for Preceptors

(Modified questions from the Research & Evaluation Team, OIT, University of Minnesota)

**Introductory text:**

This focus group is part of ongoing research on the use of iPad in the adoption of the revised family medicine curriculum and in its feasibility for use in clinical teaching environments. No one from the Department of Family Medicine will have access to the data from this session except for Doug Archibald. Your names will not be used in any reporting of the data. The conversation will be recorded and transcribed verbatim. Direct quotes and pseudonyms will be used in reporting of the data.

So we hope you will be entirely honest as you express your views about the iPad and its use. As we talk, please think about the teaching and learning in which the iPad was used, either in the clinical environment or outside.

================================================

**Questions**

**Topic 1: Usefulness:**

**Overall, was the iPad helpful to you in your academic work? Has this changed since June?**

If so, in what ways? For instance:

o Did the iPad allow you to become more familiar with the curriculum?

o Did it increase your interest in the curriculum? Did it help you to connect with your

resident?

o Did it make your teaching more efficient or more convenient?

o Are there any examples that stand out in your mind of times when the iPad was really very helpful to you (or not)?

- When is the iPad most useful, when you’re working alone, or with others?
- When is the iPad most useful, when you’re a consumer (retrieving information) or a producer (producing information)? Or is it about equally useful?

**Topic 2: The iPad and other technologies:**

The iPad is a new technology and it isn’t clear how it fits in with other technologies people may own, like cellphones, smart phones, laptops, etc.

**In general, how did you integrate the iPad with the other technologies in your life? Have you used the iPad differently or in more ways since June?**

- Did the iPad replace anything else you own, like your laptop?
- Does the iPad have any advantages for you over your laptop?
- In which circumstances, for what tasks, or in what places did you find yourself using your iPad?
- Did you find yourself carrying both or only one?
- Did you use your smartphone instead of the ipad for retrieving information even if you had the iPad with you?

**Topic 3: Problems and difficulties:**

**Overall, did you encounter a lot of problems in your use of the iPad?**

- Is there something you wish you'd known or some resource you wish you'd had as you got acquainted with your iPad?
- Do you feel that you’ve tapped the full potential of the iPad to help you learn? How do you

think you might be able to take advantage of it in the future?

**Closing**

**Would you recommend adoption of the iPad across the Department based on your experiences during the pilot?**
